# Effects of consecutive monoculture of *Pseudostellaria heterophylla* on soil fungal community as determined by pyrosequencing

**DOI:** 10.1038/srep26601

**Published:** 2016-05-24

**Authors:** Linkun Wu, Jun Chen, Hongmiao Wu, Juanying Wang, Yanhong Wu, Sheng Lin, Muhammad Umar Khan, Zhongyi Zhang, Wenxiong Lin

**Affiliations:** 1 College of Life Sciences, Fujian Agriculture and Forestry University, Fuzhou 350002, Fujian, P. R. China; 2 Key Laboratory of Biopesticide and Chemical Biology, Ministry of Education, Fujian Agriculture and Forestry University, Fuzhou 350002, Fujian, P. R. China; 3 Fujian Provincial Key Laboratory of Agroecological Processing and Safety Monitoring, College of Life Sciences, Fujian Agriculture and Forestry University, Fuzhou 35002, Fujian, P. R. China; 4 College of Crop Science, Fujian Agriculture and Forestry University, Fuzhou 350002, Fujian, P. R. China

## Abstract

Under consecutive monoculture, the biomass and quality of *Pseudostellaria heterophylla* declines significantly. In this study, a three-year field experiment was conducted to identify typical growth inhibition effects caused by extended monoculturing of *P. heterophylla*. Deep pyrosequencing was used to examine changes in the structure and composition of soil fungal community along a three-year gradient of monoculture. The results revealed a distinct separation between the newly planted plot and the two-year, three-year monocultured plots. The Shannon and Simpson diversity indices were significantly higher in the two-year and three-year monoculture soils than in the newly planted soil. Consecutive monoculture of this plant led to a significant increase in relative abundance of *Fusarium*, *Trichocladium* and *Myrothecium* and *Simplicillium*, etc., but a significant decrease in the relative abundance of *Penicillium*. Quantitative PCR analysis confirmed a significant increase in *Fusarium oxysporum*, an agent known to cause wilt and rot disease of *P. heterophylla*. Furthermore, phenolic acid mixture at a ratio similar to that found in the rhizosphere could promote mycelial growth of pathogenic *F. oxysporum*. Overall, this study demonstrated that consecutive monoculture of *P. heterophylla* can alter the fungal community in the rhizosphere, including enrichment of host-specific pathogenic fungi at the expense of plant-beneficial fungi.

*Pseudostellaria heterophylla*, which is a perennial herbaceous plant that belongs to family *Caryophyllaceae*, is highly valued in traditional Chinese medicine. *P. heterophylla* contains various active constituents, including saponins, polysaccharides, amino acids, cyclopeptides and sapogenins. This plant provides treatments for spleen deficiency, anorexia, hyperirritability, palpitation and lassitude ailments^1^. It is mainly planted in the so-called geo-authentic production region, which has the most suitable soil and climate conditions; however, consecutive monoculture of this medicinal plant in the same land leads to a significant decline in the yield and quality of the underground tubers because of increasing disease pressure, which is known as soil sickness or consecutive monoculture problem^2^. Accordingly, farmers and Chinese traditional medicine industry have made understanding the mechanism of consecutive monoculture problems exhibited in *P. heterophylla* a priority.

Many factors have been reported to cause the decline in crop yield and quality in a monoculture regime, such as soil nutrients deficiency, root exudates’ autotoxicity and imbalances in soil microbial community^3^,^4^. The autotoxicity of root exudates has been considered as one of the main reasons for consecutive monoculture problems^3^. However, previous studies frequently use the filter paper bioassays enriched with a single chemical to determine the negative effects of exuded metabolites on target plant growth, which was considered as insufficient and controversial evidence since the influences of soil chemical properties and microbial communities were excluded^5^. Therefore, the shifts of rhizospheric microbial community under monoculture have been recently attracting considerable attention^6^. As is well-known, soil ecosystem functioning is largely governed by rhizospheric microbial dynamics since microbial composition and diversity affect geochemical cycles, humus formation and degradation, soil structure and biological interactions^7^,^8^. Soil fungal communities, in terms of abundance, composition and diversity play crucial roles in the betterment of ecosystem to guarantee soil quality and crop health^9^,^10^. Wu *et al.*^11^ found that *Rehmannia glutinosa* monoculture can alter fungal community in the rhizosphere, leading to an increase in pathogenic *Fusarium oxysporum*. Zhao *et al.*^2^ successfully isolated *F. oxysporum* from infected *P. heterophylla* plants and found that the amount of *F. oxysporum* increased significantly in the rhizosphere of this plant under consecutive monoculture. However, the responses of the rhizospheric fungal community and their functional significance to consecutive monoculture of *P. heterophylla* have not yet been fully elucidated. Therefore, this study was conducted to evaluate how the abundance and composition of the soil fungal community change with the increasing years of monoculture and which soil properties contribute to shape fungal community structure.

## Results

### The morphology and yield of *P. heterophylla* under consecutive monoculture

**Figure 1:**
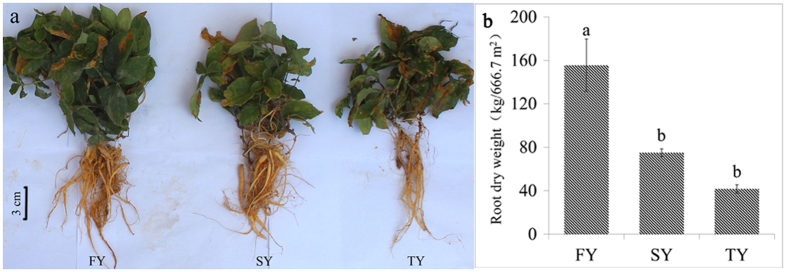
Photographs of above and below ground components of *P. heterophylla* (**a**) and changes in yield of tuber roots under consecutive monoculture (**b**). FY, SY and TY represent the newly planted, two-year and three-year monocultured *P. heterophylla*, respectively.

When compared with the newly planted plants (FY), the two-year (SY) and three-year (TY) monocultured plants displayed poorer growth with more adventitious fibrous roots and less aboveground biomass (Fig. 1a). Additionally, the dry weight of *P. heterophylla* tuber roots, the part most useful for traditional Chinese medicine, was significantly (*P* < 0.05) higher in the newly planted plots (FY) than in the two-year (SY) and three-year (TY) monocultured plots (Fig. 1b).

### Soil chemical properties of different treatments

Compared with the newly planted soil, available nitrogen (AN) and available potassium (AK) were significantly higher in the two-year monoculture soil, and all available nutrients including AN, AK and available phosphorus (AP) were significantly higher in the three-year monoculture soil. However, the newly planted soil had significantly higher total nitrogen (TN) than the two-year and three-year monoculture soils. Moreover, total potassium (TK), TP and AP were significantly higher in the newly planted soil than in the two-year monoculture soil (Table 1).

### OTU cluster and species annotation

**Figure 2:**
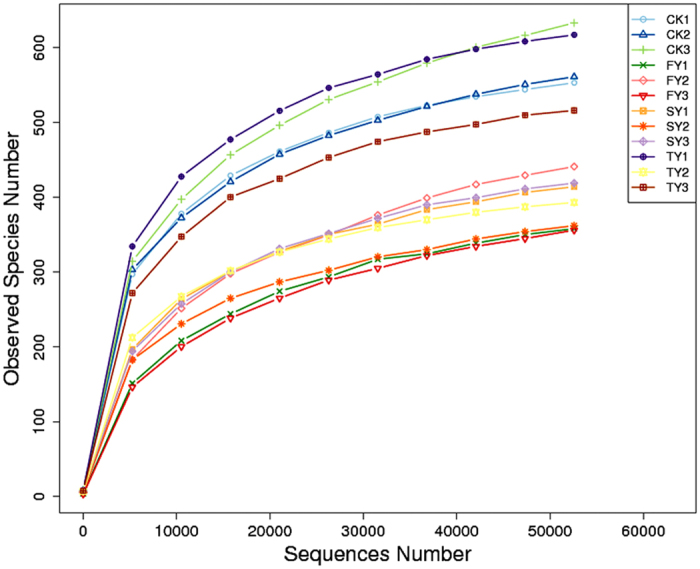
Rarefaction curves of fungal communities based on observed OTUs at 97% sequence similarity for individual samples. CK, FY, SY and TY represent the control with no *P. heterophylla* cultivation, the newly planted, two-year monocultured and three-year monocultured plots, respectively.

ITS2 deep pyrosequencing was applied to assess the effects of consecutive monoculture of *P. heterophylla* on soil fungal community. In total, 756,116 ITS2 effective tags with species annotation were obtained from 12 soil samples, with each providing an average of 63,010 effective tags (Fig. S1). Rarefaction analyses showed that the observed species number tended to plateau at 50,000 sequences (Fig. 2). Sequences from 12 soil samples were assigned to 6,242 OTUs at the 97% similarity cut-off level. There were 628, 442, 457 and 554 OTUs in CK, FY, SY and TY, respectively (Fig. S1). On average, about 99.6% of the effective sequences could be grouped to the phylum level, and more than 94% were grouped to the species level (Fig. S2).

### Alpha diversity indices

The richness and diversity indices of the fungal community in different soil samples were all calculated based on 52,585 sequences. Shannon and Simpson diversity indices were significantly higher in SY and TY than in FY (*P* < 0.05). The observed species and Chao1 were higher in SY and TY than in FY, but the difference was not significant (*P* > 0.05) (Table 2).

### Beta diversity indices

**Figure 3:**
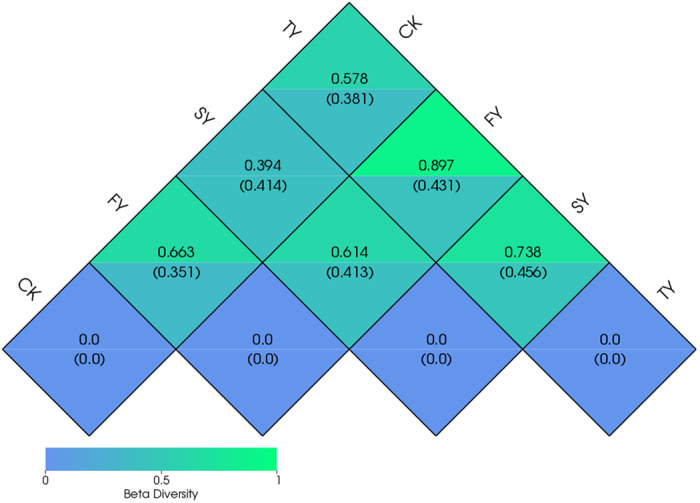
Weighted and unweighted Unifrac distances between different samples. Values in the upper and down corners of grids represented the weighted Unifrac and unweighted Unifrac distances, respectively. CK, FY, SY and TY represent the control with no *P. heterophylla* cultivation, the newly planted, two-year monocultured and three-year monocultured plots, respectively.

The weighted Unifrac and unweighted Unifrac distances between FY and SY were 0.614 and 0.413, respectively, while they were 0.897 and 0.431 between FY and TY, respectively (Fig. 3). In comparison with FY, both weighted and unweighted Unifrac distances increased with the extended monoculture.

### PCoA and UPGMA clustering

**Figure 4:**
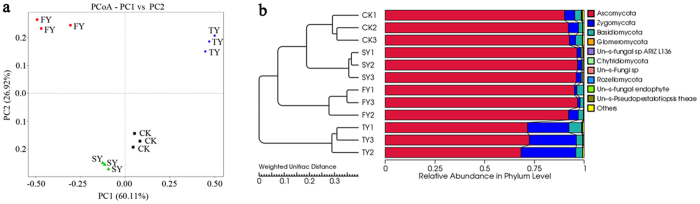
Principal coordinate analysis (PCoA) (**a**) and hierarchical clustering (**b**) of fungal communities based on weighted Unifrac algorithm for four different soil samples. CK, FY, SY and TY represent the control with no *P. heterophylla* cultivation, the newly planted, two-year monocultured and three-year monocultured plots, respectively.

Both PCoA analysis and hierarchical cluster analysis revealed distinct differences in fungal community structure among different treatments and similar patterns for the same treatments when analyzed in triplicate (Fig. 4). Furthermore, PCoA analysis showed the fungal communities in FY and SY were separated from that in TY by principle component 1, and the community in FY was separated from that in SY by principle component 2 (Fig. 4b).

### Shifts in soil fungal community composition under consecutive monoculture

The fungal OTUs were comprised mainly of six phyla, *Ascomycota*, *Zygomycota*, *Basidiomycota*, *Glomeromycota*, *Chytridiomycota* and *Rozellomycota*. *Ascomycota* was the dominant microbial taxa, accounting for 91.6%, 94.6%, 96.4% and 70.7% of the total population in CK, FY, SY and TY, respectively (Fig. S3).

**Figure 5:**
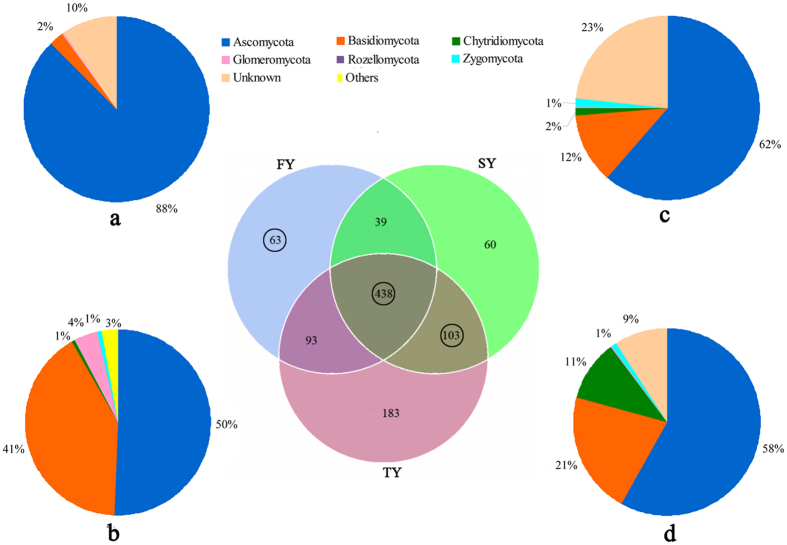
Venn diagram of exclusive and shared species-level taxa among the newly planted (FY), two-year monocultured (SY) and three-year monocultured (TY) soils. (**a**) Pie chart of shared OTUs (438) among 3 time-scale soil samples based on the average abundance of each phylum in FY, SY and TY. (**b**) Pie chart of exclusive OTUs (63) in FY; (**c**) Pie chart of exclusive OTUs (103) shared between SY and TY based on the relative abundance of each phylum in SY. (**d**) Pie chart of exclusive OTUs (103) shared between SY and TY based on the relative abundance of each phylum in TY.

The percentage of species-level taxa shared in FY, SY and TY was 44.7% (438 species). Most species belongs to the phylum *Ascomycota* (88%) (Fig. 5a). The number of OTUs exclusively found in FY samples was 63 (6.4%), and these were mainly assigned to the phyla *Ascomycota* (50%) and *Basidiomycota* (44%) (Fig. 5b). The number of exclusive OTUs shared between SY and TY was 103 (10.5%), showing the similar community structure in SY and TY (Fig. 5c,d).

**Figure 6:**
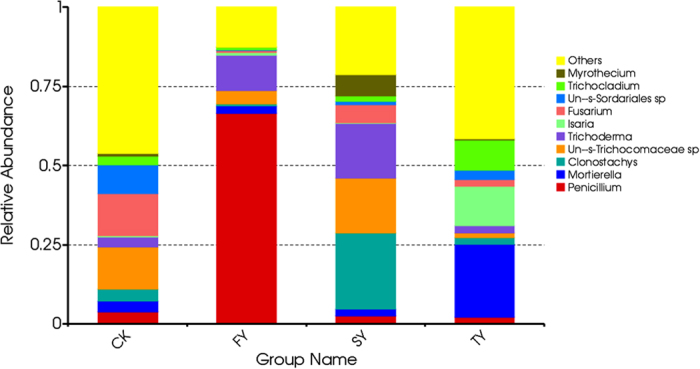
Relative abundances of the top 10 fungal genera in four different soil samples. CK, FY, SY and TY represent the control with no *P. heterophylla* cultivation, the newly planted, two-year monocultured and three-year monocultured plots, respectively. The prefix ‘Un-s-’ means the OTUs were unidentified in the Unite Database.

Furthermore, at fungal genus level, consecutive monoculture of this plant led to a significant increase in the relative abundances of *Fusarium*, *Clonostachys*, *Mortierella*, *Trichocladium* and *Myrothecium*, but a significant decrease in the relative abundance of *Penicillium* (Fig. 6). Pathogenic *F. oxysporum* was frequently isolated from consecutively monocultured soil and infected *P. heterophylla* (Fig. S4). A *F. oxysporum* strain isolated from the infected plant parts could rapidly cause wilt disease on the seedlings of *P. heterophylla* in both tissue culture vessels under sterile conditions and in pots with sterilized soils (Fig. S4). Compared with NP, the genus *Trichoderma* increased in SY but decreased in TY.

**Figure 7:**
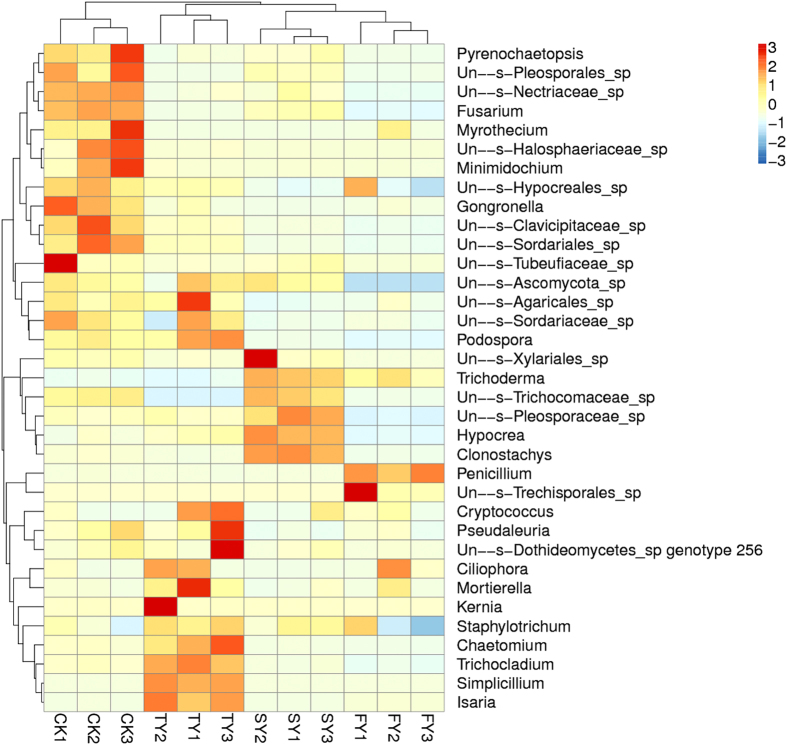
Heat map analysis of the top 35 most abundant genera in four different treatments. CK, FY, SY and TY represent the control with no *P. heterophylla* cultivation, the newly planted, two-year monocultured and three-year monocultured plots, respectively. The prefixes ‘Un-s-’ and ‘IS-s-’ mean unidentified OTUs and incertae sedis taxa in the Unite Database, respectively.

Heat map analysis of the top 35 most abundant genera within a hierarchical cluster showed obvious variations in fungal community structure across the three time-scale replanted soils. Moreover, when compared with FY, the difference in community structure increased with increasing years of monoculture, indicating a gradual shift in the rhizosphere fungal community following extended monoculture (Fig. 7). When SY and TY were combined as one sample and termed “consecutively monocultured soil (CM)”, SIMPER analysis (relative abundance of genera, logarithmic transformation and standardization) showed that the pairwise dissimilarity in the fungal community between FY and CM was 52.5%. The top genera with 40% cumulative contribution to the dissimilarity between FY and CM are listed in Table 3, and these included *Penicillium*, *Fusarium*, *Isaria*, *Myrothecium* and *Simplicillium*, etc. (Table 3).

### Effects of soil chemical properties on dominant genera

**Figure 8:**
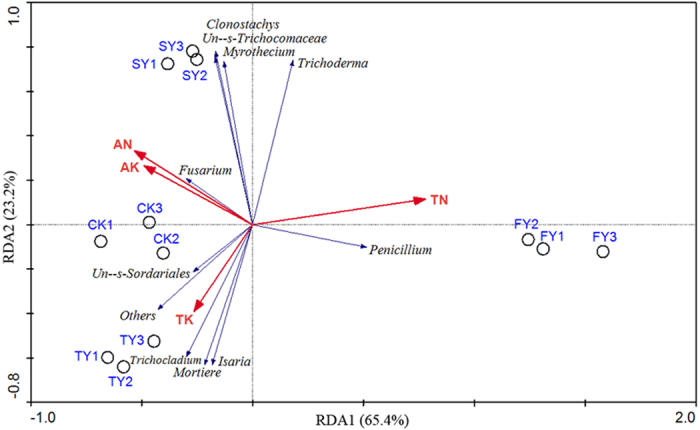
Redundancy analysis (RDA) of dominant genera and soil chemical properties for individual sample from four different treatments. CK, FY, SY and TY represent the control with no *P. heterophylla* cultivation, the newly planted, two-year monocultured and three-year monocultured plots, respectively. TN and AN represent total and available nitrogen, respectively. TK and AK represent total available potassium, respectively.

Redundancy analysis (RDA) of the dominant genera data and soil chemical properties revealed remarkable variations in fungal community structure in the three time-scale replanted soils. The first two RDA components (RDA1 and RDA2) could explain 65.4% and 23.2% of the total variance, respectively. The first RDA component (RDA1) separated the newly planted soils (NP) from the two-year (SY) and three-year (TY) monocultured soils. The newly planted soil was positively related to the higher relative abundance of *Penicillium*, and the higher contents of total N (TN), but negatively related to *Fusarium*, as well as higher contents of available N (AN) and available K (AK) (Fig. 8). A forward selection of environmental variables was carried out by a Monte-Carlo permutation test. Variables including total N (*P* = 0.02), available N (*P* = 0.04), total K (*P* = 0.02) and available K (*P* = 0.02) were significantly correlated with variations in the fungal community structure. The relative abundance of *Penicillium* was positively correlated with soil total N and negatively correlated with soil available N and available K. In contrast, the relative abundance of *Fusarium* was positively correlated with soil available N and available K and negatively correlated with soil total N (Fig. 8).

### Abundance of *F. oxysporum* by quantitative PCR

**Figure 9:**
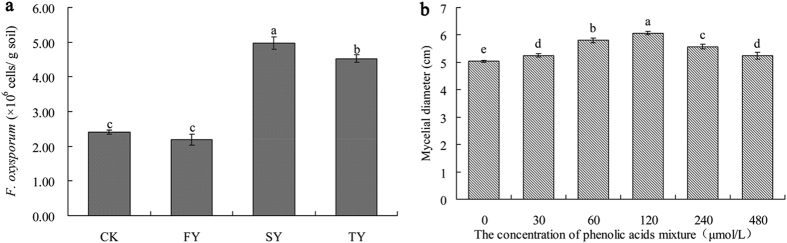
Quantification of *F. oxysporum* in four different soil samples (**a**) and effects of phenolic acids on *F. oxysporum* growth (**b**). CK, FY, SY and TY represent the control with no *P. heterophylla* cultivation, the newly planted, two-year monocultured and three-year monocultured plots, respectively.

The amount of *F. oxysporum* was significantly greater in consecutive monoculture soils (SY and TY) than in newly planted soils (FY) (Fig. 9a). The result was consistent with the deep pyrosequencing analysis except for the control. Moreover, a phenolic acid mixture at the same ratio found in the *P. heterophylla* rhizosphere could significantly promote the mycelial growth of isolated *F. oxysporum*. The mycelium diameter increased as the concentration increased until reaching a plateau at 120 μmol/L concentration (Fig. 9b).

## Discussion

Consecutive monoculture problem, also known as replant disease or soil sickness, is common to Chinese medicinal herbs in all growing regions. About 70% of medicinal plant species with tuber roots have various degrees of consecutive monoculture problems, including *P. heterophylla*, *Rehmannia glutinosa*, and *Panax notoginseng*, etc^12^. Our three-year field experiment revealed typical growth inhibition effects caused by consecutive monoculture of *P. heterophylla*, with poor plant performance and insufficient resistance for disease and pests (Fig. 1). Studies of consecutive monoculture problems have centered on nutrient availability and the autotoxicity of allelochemicals released by roots^3^,^4^. However, increasing studies have shown that soil available nutrients did not decrease under consecutive monoculture of medicinal plants and fertilization was not effective for eliminating replant disease^13^,^14^. In the current work, we also found that some available nutrients including AN, AK and AP were significantly higher in the consecutively monocultured soils than in the newly planted soil (Table 1). Besides, many researchers have begun to doubt that allelochemicals are present in sufficient concentrations in soil to directly affect the growth of neighboring plants or the host plant^5^,^15^,^16^. If they could have these effects, the observation that replant disease can endure for many years after harvest would imply that allelochemicals are extraordinarily resilient to the degradation by soil microbes^17^. In our previous study, we found that most phenolic acids in rhizosphere soil of *P. heterophylla* did not continuously accumulate with increasing years of monoculture^18^. Moreover, mixtures of these phenolic acids at the same ratio found in the rhizosphere soil did not show direct autotoxicity toward tissue culture seedlings of *P. heterophylla*^18^.

Recently, the roles of belowground microbial community in aboveground plant performance have been of increasing interest^6^,^19^,^20^. Microbes are the unseen majority in soil and act as the most important drivers of plant health and productivity^21^,^22^. Fungi, an important group of microbes in the soil ecosystem, are crucial for soil functions and plant health^23^,^24^,^25^. In this study, pyrosequencing of ITS2 amplicons showed great shifts in fungal community composition and diversity in the rhizosphere after consecutive monoculture of *P. heterophylla*. The Shannon and Simpson diversity indices were significantly higher in the two-year and three-year monoculture soils than in the newly planted soil (Table 2). Xiong *et al.*^26^ found that soil fungal diversity index including the Chao1, ACE and Shannon index increased significantly after monoculture of vanilla (*Vanilla planifolia*). Zhou *et al.*^27^ found that exogenously applied *p*-hydroxybenzoic acid, an autotoxin of cucumber, increased the Shannon-Wiener index of the fungal community in the rhizosphere. Furthermore, we found that consecutive monoculture of this plant led to a significant increase in the relative abundances of *Fusarium*, *Myrothecium* and *Simplicillium*, etc., but a significant decrease in the relative abundance of *Penicillium* (Table 3). Among them, *Fusarium* spp. are well-known pathogens for many important crops^28^. The pathogenic fungi *F. oxysporum*, which is an agent of wilt and rot disease of *P. heterophylla*, was frequently isolated from infected tuber roots of this plant^2^. Quantification analysis by qPCR in this study further confirmed a significant increase in *F. oxysporum* in rhizosphere soil after *P. heterophylla* monoculture (Fig. 9). Moreover, *Myrothecium* spp. and *Simplicillium* spp., which have been reported to be pathogens of a number of plants^29^,^30^, exhibited a higher relative abundance in SY or TY than in FY (Table 3). However, the relative abundance of genus *Penicillium* decreased from 66.47% in FY to 2.63% in SY and 2.10% in TY (Table 3). Although some *Penicillium* species can cause plant diseases^31^, many of them are reportedly plant beneficial fungi that could enhance protection against pathogens by induced systemic resistance (ISR) and promote plant growth^32^,^33^. Hossain *et al.*^34^ demonstrated that the plant-growth-promoting-fungus *Penicillium* sp. GP16-2 could induce systemic resistance against *Pseudomonas syringae* in *Arabidopsis thaliana*. In this study, the decrease of genus *Penicillium* from FY to SY and TY was mainly attributed to the decrease of *Penicillium* sp. P_33. The total abundance of other *Penicillium* species (including *Penicillium guanacastense*, *Penicillium oxalicum*, *Penicillium* sp. MI 31 and *Penicillium sumatrense*) accounted for less than 2% of the total in the four soils. *Penicillium* sp. P_33 (NCBI Taxonomy ID: 472018, http://www.ncbi.nlm.nih.gov/nuccore/157419957) has been reported to possess antagonistic activity against plant pathogen *Phytophthora citricola*. These results suggest that the replant disease of *P. heterophylla* might be attributed to the rapid proliferation of potential pathogens at the expense of plant-growth-promoting-fungi in replanted soil. Li *et al.*^28^ found that fungal pathogens accumulated at the expense of plant-beneficial fungi under consecutive monoculture of peanut (*Arachis hypogaea* L.). In addition, many studies reported that the consecutive monoculture problems of plants resulted from shifts in the soil microbial community induced by root exudates rather than direct allelopathic autotoxicity^11^,^35^,^36^. Increasing studies have shown that root exudates could select microorganisms in the rhizosphere, and that these plant-associated microorganisms could then influence plant growth and health^37^,^38^,^39^. In this study, a phenolic acid mixture at the same ratio detected in the *P. heterophylla* rhizosphere could significantly promote the mycelial growth of pathogenic *F. oxysporum*. Zhou *et al.*^40^ found *p*-coumaric acid detected in root exudates of cucumber could significantly increase the densities of pathogenic *F. oxysporum* f.sp. *cucumerinum* Owen in soil. Moreover, Li *et al.*^35^ found peanut root exudates could selectively inhibit or stimulate certain microorganisms, particularly, the relative abundance of *F. oxysporum*. However, it should be noted that the positive effects of root-released phenolic acids on *F. oxysporum* may not be limited to the promotion of mycelial growth, but also include promotion of spore germination, toxin production and the epigenetic regulation of gene expression. Moreover, other root exudates such as soluble sugar, organic acids, and amino acids might also play important roles in the growth of *F. oxysporum*. All of these factors may contribute to the proliferation of pathogens. Accordingly, more researches in these aspects are needed and are currently underway.

In conclusion, consecutive monoculture of *P. heterophylla* can alter the fungal communities in the rhizosphere, resulting in fewer plant-beneficial fungi and more pathogenic fungi, which may be a contributing factor to soil sickness of this plant. However, further work is needed to explore the underlying mechanisms of the rhizosphere interactions between pathogenic and beneficial microorganisms and the roles of root exudates in the rhizosphere. The isolation of specific microorganisms (i.e., *Penicillium* spp. and *Fusarium* spp.) and their relationships with replant disease of this plant would also help understand this phenomenon.

## Materials and Methods

### Field experiment and soil sampling

*P. heterophylla* cultivar ‘Zheshen 2’, which is common in the main production region, was used as the test plant material. *P. heterophylla* was planted on November 20 and harvested by June 30 of the following year. After harvest, fields were kept fallow from July 1 to November 19. The experiment was conducted at Fuding City, Fujian Province (27°26′N, 120°04′E), which has a subtropical oceanic monsoon climate, annual mean temperature of 18.4 °C and an annual mean precipitation of 1668.3 mm. A field previously cultivated with *Oryza sativa* was used for this experiment with four treatments: i) the newly planted (FY), ii) two-year consecutive monoculture (SY), iii) three-year consecutive monoculture (TY) and iv) control with no *P. heterophylla* cultivation (CK). The soil chemical properties before the experiment were as follows: total nitrogen 1.83 g · kg^−1^, total phosphorus 0.47 g · kg^−1^, total potassium 8.46 g · kg^−1^, available nitrogen 26.23 mg · kg^−1^, available phosphorus 96.34 mg · kg^−1^, available potassium 365.21 mg · kg^−1^, pH 5.32. All treatments were organized within a single field site to keep the same soil and climatic conditions and subjected to the same fertilization and field management during the experimental period. Each treatment had three replicate plots and the study plots were completely randomized.

On April 15, 2014 we collected soil samples from 5 random locations within each plot because of the significant difference in growth status between different treatments on this date. The rhizosphere soil that clung to roots and rhizomes of *P. heterophylla* was brushed off and collected. Soil samples were sieved through 2 mm mesh to immediately extract total soil DNA. The rest of the soil samples were air-dried and used to determine soil physical–chemical properties^13^.

### DNA extraction and PCR amplification

Total DNA were extracted from soil samples using SoilGen DNA kit (CWBIO, Beijing, China) following the manufacturer’s instructions. DNA concentration was determined by using Nanodrop 2000C Spectrophotometer (Thermo Scientific, USA) and then DNA was diluted to 1 ng/μL using sterile water. Internal transcribed spacer (ITS2) was amplified by using specific primer ITS2F (GCATCGATGAAGAACGCAGC) and ITS2R (TCCTC CGCTTATTGATATGC) with the barcode. All PCR reactions were carried out with Phusion High-Fidelity PCR Master Mix (New England Biolabs, Ipswich, USA).

### PCR purification and pyrosequencing

PCR products were monitored on 2% agarose gel and samples with bright main strip between 400–450 bp were chosen for further analysis. PCR products were mixed at an equal ratio and purified with Qiagen Gel Extraction Kit (Qiagen, Hilden, Germany). Sequencing libraries were generated using TruSeq DNA PCR-Free Sample Preparation Kit (Illumina, San Diego, USA) following the manufacturer’s instructions. Pyrosequencing was performed on an Illumina HiSeq2500 platform and 250 bp paired-end reads were generated.

### OTU (Operational Taxonomic Unit)-based sequence analysis

The sequence reads were assigned to each sample based on their unique barcode and truncated by cutting off the barcode and primer sequences. Paired-end reads were merged using FLASH (V1.2.7)^41^. Through quality filtering and chimera removal, the retained sequences called as effective tags were used to perform OTU cluster and species annotation. Sequences with ≥97% similarity were assigned to the same OTU through Uparse software (Uparse v7.0.1001)^42^. Species annotation was carried out via the Unite Database (https://unite.ut.ee/)^43^ based on Blast algorithm which was calculated by QIIME software (Version 1.7.0).

### Quantitative PCR for *F. oxysporum*

Quantitative PCR (qPCR) was carried out to quantify *F. oxysporum* in different soil samples by using the primers ITS1-F (CTTGGTCATTTAGAGGAAGTAA) and AFP308R (CGAATTAACGCGAGTCCCAA)^44^. Quantitative PCR was performed in 15 μl reaction mixture containing 7.5 μl 2× SYBR green I SuperReal Premix (TIANGEN, Beijing, China), 0.5 μl of each primer (10 μM) and template DNA (20 ng of total soil DNA or a serial dilution of plasmid DNA for standard curves). Four independent quantitative PCR assays were performed for each treatment.

### The effects of phenolic acids on the growth of isolated *F. oxysporum*

Based on the HPLC results of phenolic acids in the *P. heterophylla* rhizosphere^18^, a phenolic acid mixture at the same ratio as detected in soil including gallic acid, coumaric acid, *p*-hydroxybenzoic acid, vanillic acid, syringic acid, vanillin, ferulic acid and benzoic acid was used to assess the effects on the growth of isolated *F. oxysporum*. Specifically, the isolated *F. oxysporum* was inoculated onto the center of a 9 cm diameter Petri dish filled with a 10-fold dilution of soil-extract agar medium (SEM) containing different final concentrations of phenolic acid mixtures (30, 60, 120, 240 and 480 μmol/L). Each treatment had three replicates. The mycelium diameter was measured after eight days of incubation at 28 °C in 0:24 under a 0:24 light: dark cycle.

### Statistical analyses

The abundances of OTUs were normalized, after which alpha and beta diversity analyses were performed based on the normalized data. Alpha diversity was applied to analyze species complexity for a sample through six indices, observed-species, community richness indices (chao1, ACE) and diversity indices (Shannon’s, Simpson’s). Beta diversity analysis was used to evaluate differences in species complexity between samples, including principal coordinate analysis (PCoA) and unweighted pair-group method with arithmetic means (UPGMA) clustering.

For all parameters, one way analysis of variance (ANOVA) followed by Tukey’s test (*P* < 0.05) was used for multiple comparisons through DPS software version 7.51. Similarity percentage analysis (SIMPER) for assessing the relative contribution (%) of each microbial taxa to the dissimilarity between samples was performed with the PRIMER V5 software package (PRIMER-E Ltd, Plymouth, UK)^45^.

## Additional Information

**How to cite this article**: Wu, L. *et al.* Effects of consecutive monoculture of *Pseudostellaria heterophylla* on soil fungal community as determined by pyrosequencing. *Sci. Rep.* **6**, 26601; doi: 10.1038/srep26601 (2016).

## Supplementary Material

Supplementary Information

## Figures and Tables

**Table 1:** Chemical properties of soils from four different treatment plots.

Treatments	TN (g/kg)	AN (mg /kg)	TP (g/kg)	AP (mg/kg)	TK (g/kg)	AK (mg/kg)
CK	1.78c	184.89b	0.28c	76.47b	8.20b	153.66c
FY	2.74a	165.81d	0.34a	77.34b	8.21b	134.73d
SY	2.13b	189.42a	0.31b	70.61c	8.02c	219.74a
TY	1.75c	172.36c	0.34a	106.09a	9.23a	183.18b

TN, AN, TP, AP, TK, AK represent total nitrogen, available nitrogen, total phosphorus, available phosphorus, total potassium and available potassium, respectively. CK, FY, SY and TY represent the control with no *P. heterophylla* cultivation, the newly planted, two-year monocultured and three-year monocultured plots, respectively. Different letters in columns show significant differences determined by Tukey’s test (*P* ≤ 0.05, n = 3).

**Table 2:** Calculations of observed species, richness and diversity in different soil samples.

Treatments	Observed species	Chao1	ACE	Shannon	Simpson
CK	582.33a	665.29a	673.23a	5.85a	0.96a
FY	385.00b	452.54b	466.79a	2.47c	0.54b
SY	398.33b	455.20ab	459.44a	4.32b	0.88a
TY	508.67ab	550.65ab	550.27a	5.13ab	0.91a

CK, FY, SY and TY represent the control with no *P. heterophylla* cultivation, the newly planted, two-year monocultured and three-year monocultured plots, respectively. Different letters in columns show significant differences determined by Tukey’s test (*P* ≤ 0.05, n = 3).

**Table 3:** Top genera with 50% cumulative contribution to the dissimilarity between the newly planted and consecutively monocultured soils.

Genus	Class	Phylum	Contribution	Relative abundance (%)			
			(%)	CK	FY	SY	TY
*Penicillium*	*Eurotiomycetes*	*Ascomycota*	9.73	3.85b	66.47a	2.63b	2.10b
*Clonostachys*	*Sordariomycetes*	*Ascomycota*	5.87	3.77b	0.59c	24.10a	2.11bc
*Mortierella*	IS–s-*Mortierella* sp	*Zygomycota*	3.8	3.38b	2.37b	2.02b	23.12a
*Fusarium*	*Sordariomycetes*	*Ascomycota*	3.78	13.30a	0.49d	5.77b	2.18c
*Isaria*	*Sordariomycetes*	*Ascomycota*	3.75	0.28b	0.82b	0.17b	12.33a
*Myrothecium*	*Sordariomycetes*	*Ascomycota*	3.53	0.72b	0.15b	6.76a	0.38b
*Trichocladium*	*Sordariomycetes*	*Ascomycota*	3.48	2.79b	0.78c	1.79bc	9.44a
Un–s-*Trichocomaceae* sp	*Eurotiomycetes*	*Ascomycota*	3.33	13.30b	4.26c	17.21a	1.43d
*Simplicillium*	*Sordariomycetes*	*Ascomycota*	3.09	0.12b	0.05b	0.09b	6.97a

CK, FY, SY and TY represent the control with no *P. heterophylla* cultivation, the newly planted, two-year monocultured and three-year monocultured plots, respectively. The prefixes ‘Un-s-’ and ‘IS-s-’ mean unidentified OTUs and incertae sedis taxa in the Unite Database, respectively. Different letters in columns show significant differences determined by Tukey’s test (*P* ≤ 0.05, n = 3).
