# Supplementary material for: Effects of consecutive monoculture of Pseudostellaria heterophylla on soil fungal community as determined by pyrosequencing
Source: Sci Rep. 2016 May 24;6:26601. doi: 10.1038/srep26601 (PMC4877567; doi:10.1038/srep26601)
Supplement: Supplementary Information [file srep26601-s1.doc]

**Effects of Consecutive Monoculture of *Pseudostellariae heterophylla* on** **Soil Fungal Community as Determined by** **Pyrosequencing**

Linkun Wu^1,2*^, Jun Chen^1,2*^, Hongmiao Wu^1,2^, Juanying Wang^1,2^, Yanhong Wu^1,2^, Sheng Lin^1,3^, Muhammad Umar Khan^1,3^, Zhongyi Zhang^3,4^ & Wenxiong Lin^1,3^,^**^

^1^College of Life Sciences, Fujian Agriculture and Forestry University, Fuzhou 350002, Fujian, P. R. China.

^2^Key Laboratory of Biopesticide and Chemical Biology, Ministry of Education, Fujian Agriculture and Forestry University, Fuzhou 350002, Fujian, P. R. China.

^3^Fujian Provincial Key Laboratory of Agroecological Processing and Safety Monitoring, College of Life Sciences, Fujian Agriculture and Forestry University, Fuzhou 35002, Fujian, P. R. China.

^4^College of Crop Science, Fujian Agriculture and Forestry University, Fuzhou 350002, Fujian, P. R. China.

^**^Correspondence and requests for materials should be addressed to W.X.L. (email: lwx@fafu.edu.cn).

^*^These authors contributed equally to this work.

**Additional information**

**Figure S1** **| Statistics of OTU cluster and species annotation for individual samples.** Total Tags (red columns): represent the total number of effective tags; Unique Tags (orange columns): represent the total numbers of singletons which were removed from the dataset before further analysis; Taxon Tags (blue columns): represent the total numbers of tags subjected to OTU cluster and with species annotation; Unclassified Tags (green coclumns): represent the total numbers of tags without species annotation; OTUs (purple columns): represent the OTUs numbers for each sample. CK, FY, SY and TY represent the control with no *P. heterophylla* cultivation, the newly planted, two-year monocultured and three-year monocultured plots, respectively.

**Figure S2 | Sequence numbers for** **each taxonomic level in 12 different soil samples.** CK, FY, SY and TY represent the control with no *P. heterophylla* cultivation, the newly planted, two-year monocultured and three-year monocultured plots, respectively.

**Figure S3 | Relative abundances of the top 10 fungal phyla in four different soil samples.** CK, FY, SY and TY represent the control with no *P. heterophylla* cultivation, the newly planted, two-year monocultured and three-year monocultured plots, respectively. The prefix ‘Un-s-’ means the OTUs were unidentified in the Unite Database.

**Figure S4 | Morphology o of isolated *F. oxysporum* (a) and its pathogenicity assessment (b, c).** CK represents the control without *F. oxysporum* inoculation; FON represents the treatment inoculated with *F. oxysporum.*


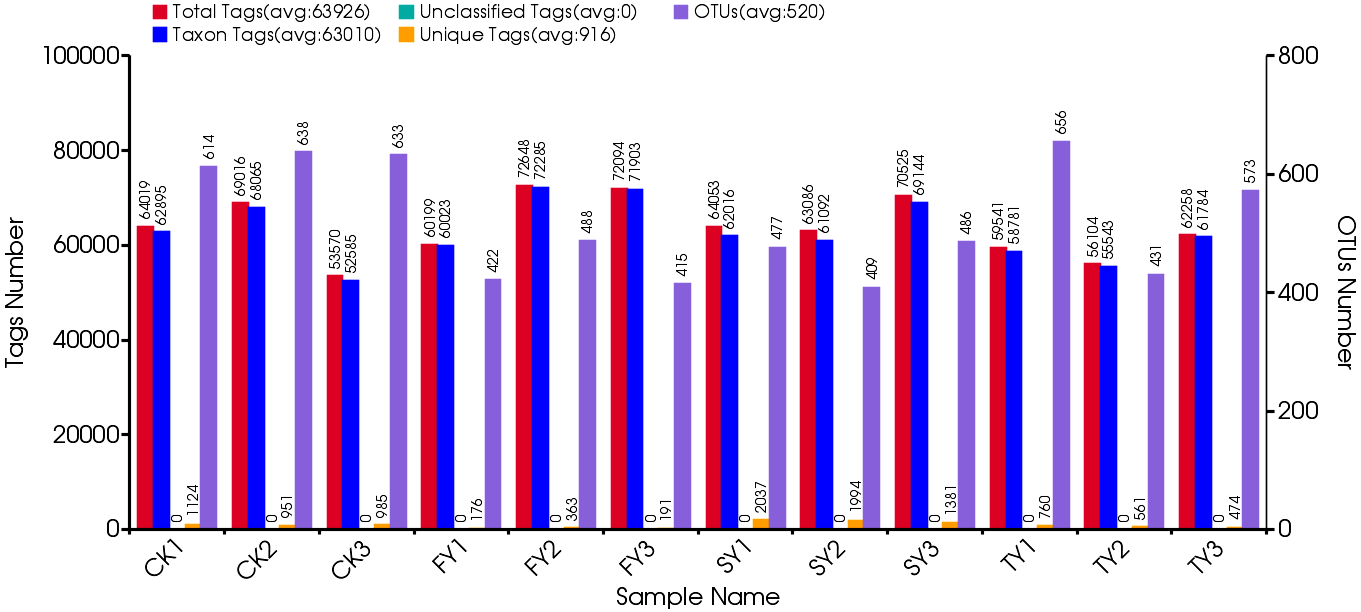


**Figure S1 | Statistics of OTU cluster and species annotation for individual samples.** Total Tags (red columns): represent the total numbers of effective tags; Unique Tags (orange columns): represent the total numbers of singletons which were removed from the dataset before further analysis; Taxon Tags (blue columns): represent the total numbers of tags subjected to OTU cluster and with species annotation; Unclassified Tags (green coclumns): represent the total numbers of tags without species annotation; OTUs (purple columns): represent the OTUs numbers for each sample. CK, FY, SY and TY represent the control with no *P. heterophylla* cultivation, the newly planted, two-year monocultured and three-year monocultured plots, respectively.


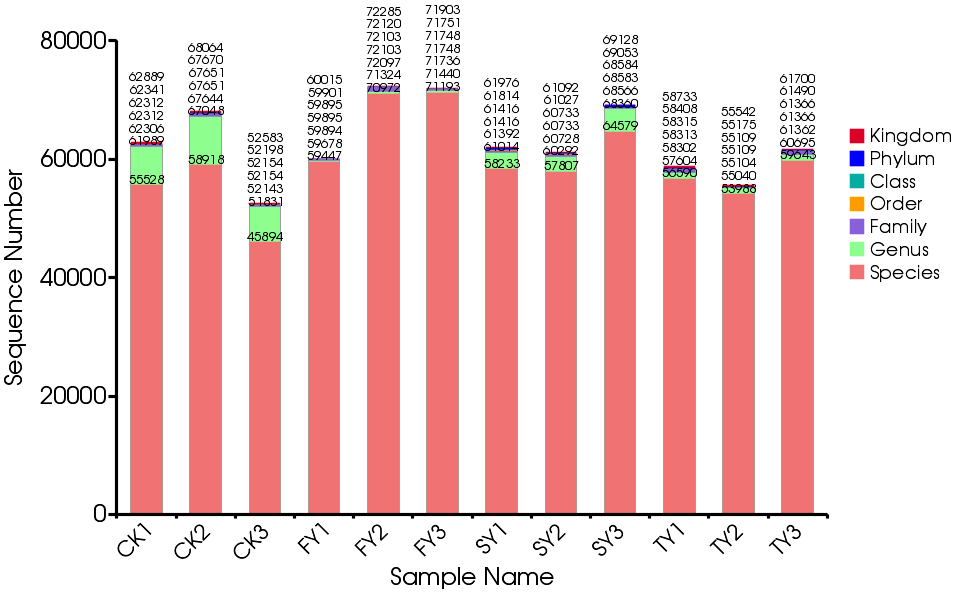


**Figure S2 | Sequence numbers for** **each taxonomic level in 12 different soil samples.** CK, FY, SY and TY represent the control with no *P. heterophylla* cultivation, the newly planted, two-year monocultured and three-year monocultured plots, respectively.


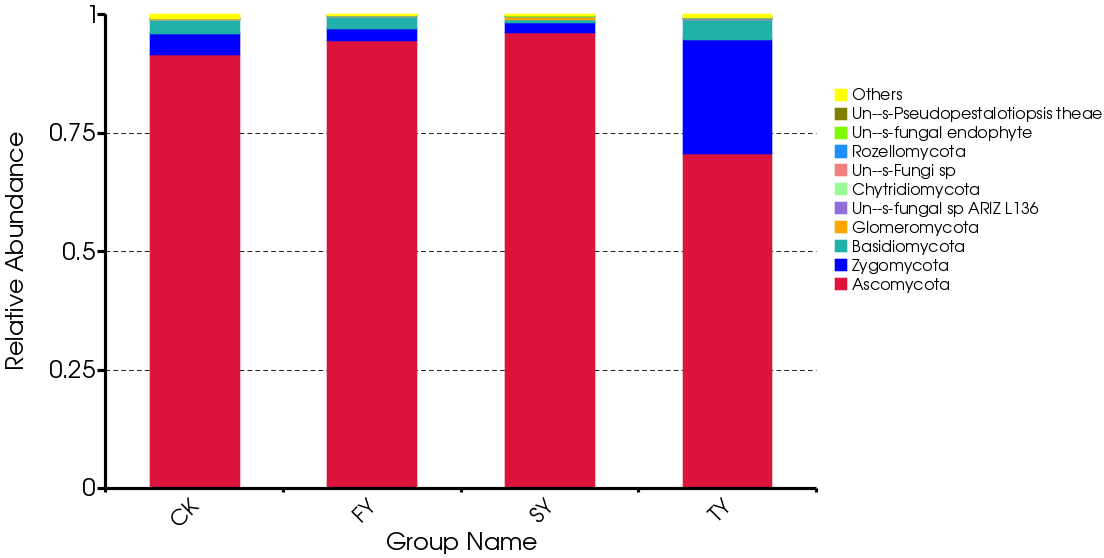


**Figure S3 | Relative abundances of the top 10 fungal phyla in four different soil samples.** CK, FY, SY and TY represent the control with no *P. heterophylla* cultivation, the newly planted, two-year monocultured and three-year monocultured plots, respectively. The prefix ‘Un-s-’ means the OTUs were unidentified in the Unite Database.


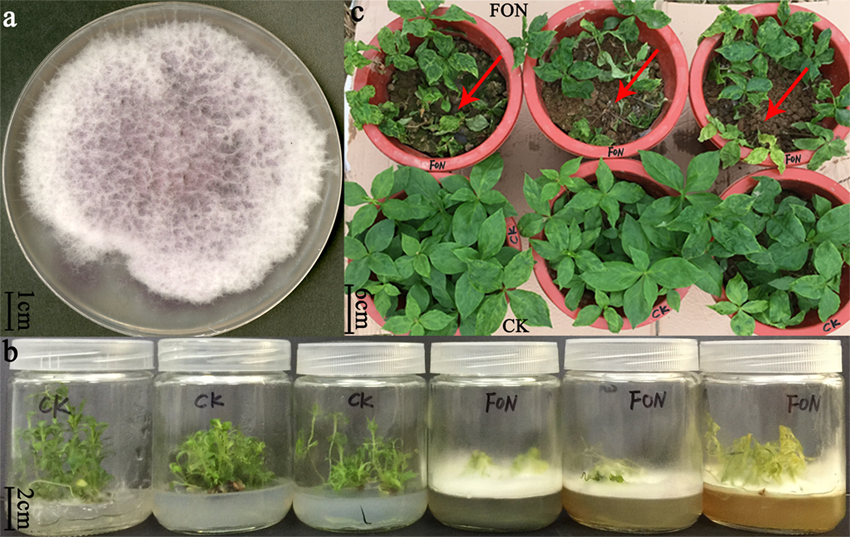


**Figure S4 | Morphology o of isolated *F. oxysporum* (a) and its pathogenicity assessment (b, c).** CK represents the control without *F. oxysporum* inoculation; FON represents the treatment inoculated with *F. oxysporum.*
